# Focal aware seizures manifesting as restless legs syndrome‐like symptoms in a patient with periodic limb movement disorder

**DOI:** 10.1002/epd2.70041

**Published:** 2025-05-23

**Authors:** Ammar Rasul, Ashar M. Farooqi, Jon D. Peters, Yong Won Cho, Gholam K. Motamedi

**Affiliations:** ^1^ Department of Pulmonary & Critical Care Medicine MedStar Georgetown University Hospital Washington District of Columbia USA; ^2^ Department of Neurology University of Kentucky College of Medicine Lexington Kentucky USA; ^3^ Department of Neurology MedStar Georgetown University Hospital Washington District of Columbia USA; ^4^ Department of Neurology Keimyung University School of Medicine Daegu South Korea

**Keywords:** focal seizure, periodic limb movement disorder, restless leg syndrome

Co‐existing sleep disorders and epilepsy can complicate the diagnosis and management of both conditions. The overlap of sensory and motor symptoms between sleep disorders—such as restless leg syndrome (RLS), periodic limb movement disorder (PLMD), and seizure‐related manifestations—can make accurate diagnosis challenging, leading to potential misdiagnosis and mismanagement. Similarly, focal aware seizures with sensory auras may be misinterpreted as primary sensory symptoms of RLS, particularly when they occur bilaterally and are described by the patient as a sensation of discomfort or restlessness. Furthermore, recent evidence suggests a strong association between epilepsy and RLS.^1^, ^2^, ^3^, ^4^ This clinical vignette highlights this complexity.

We present a 51‐year‐old female with focal aware seizures who experienced sensory symptoms typical of RLS as her ictal symptoms, as well as paroxysmal nocturnal motor symptoms meeting the diagnostic criteria for PLMD. She presented with new onset, predominantly nocturnal, stereotypical leg movements that had raised suspicion of seizures, especially after experiencing her first seizure during sleep. She also reported randomly occurring diurnal episodes of a “creepy‐crawly” sensation bilaterally in her upper and lower extremities, as well as her trunk, all with intact awareness. There was no circadian pattern to this sensation, nor an urge to move, improvement with movement, or worsening at rest to fully meet the diagnostic criteria for RLS. Upon further questioning, she also reported nonrestorative sleep and daytime sleepiness. In‐laboratory polysomnography revealed mild obstructive sleep apnea (apnea/hypopnea index 9.8/h, oxygen desaturation 88%) and moderate‐to‐severe periodic limb movement in sleep (PLMS) (43 events/hour; Figure 1A). She had a normal brain MRI, and a loop recorder had ruled out cardiac arrhythmia.

**FIGURE 1:**
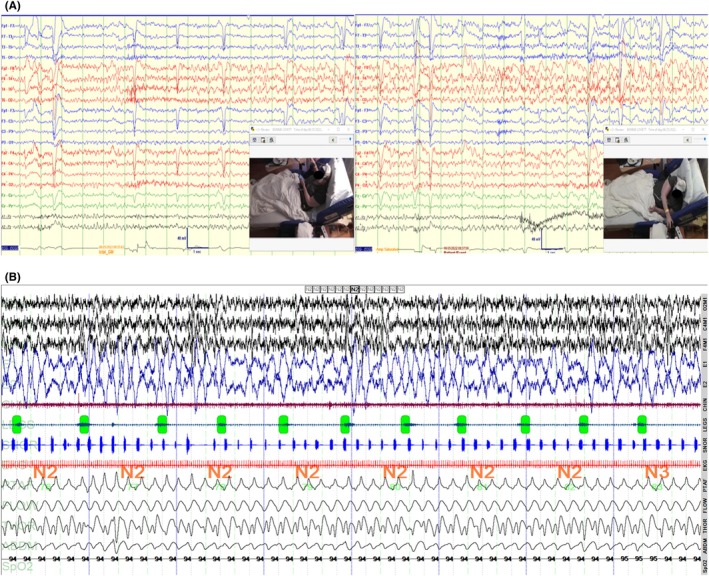
(A) Frequent periodic limb movements of sleep (PLMS); 43/hour. N2 sleep, standard polysomnogram. (B) Focal aware seizure during wakefulness: Buildup of rhythmic repetitive sharp discharges on the right, max anterior. Patient pressed the event button and reported a “creepy‐crawly” sensation in both upper and lower extremities; 30‐s recording, standard 10–20 electrode system.

During admission to the epilepsy monitoring unit, 11 focal seizures originating from the right hemisphere, maximally in the lateral anterior leads, were recorded (Figure 1B). Five seizures were nocturnal and subclinical, with no abnormal motor activity except for one that became secondarily generalized. Interestingly, during several diurnal seizures lasting ~60 s, she was able to describe her symptoms to her nurse as a “creepy‐crawly” sensation in all upper and lower extremities, spreading to her trunk, without any lateralizing symptoms or abnormal motor activity. Only one such sensory episode was recorded without associated ictal discharges.

Laboratory tests were unremarkable, except for a very low serum ferritin level (4.5 ng/mL) leading to the initiation of iron supplementation therapy. Treatment with positive airway pressure resolved her daytime sleepiness and PLMS/PLMD. A follow‐up ferritin level normalized to 46 ng/mL. After adding lamotrigine to her levetiracetam monotherapy regimen, her seizure frequency improved, and her auras changed to a sensation of tingling throughout her body. With further dose adjustments, her seizures and episodes of “creepy‐crawly” sensations completely resolved (2.5‐year follow‐up).

This case is unique in that the patient's focal aware seizures (auras) mimicked the hallmark RLS symptom of a bilateral creepy‐crawly sensation, in a patient with very low ferritin and PLMS who otherwise did not meet the full diagnostic criteria for RLS. This highlights the complex interplay between epilepsy, RLS, and PLMD, and the diagnostic challenge in patients with focal aware seizures, where patients' ability to verbally communicate during events may lead to misrecognition of seizures by the patient and family.

This case aligns with prior evidence suggesting a higher frequency of PLMS in focal epilepsy (80% vs. 4.8% in healthy controls).^4^ An association between PLMS and arousal instability in a patient with nocturnal frontal lobe epilepsy has been proposed, suggesting a potential link between the two.^5^ In the absence of stereotactic EEG recording in our patient, we could not accurately localize the seizure onset zone. RLS has also been reported as a comorbidity in patients with epilepsy, though its frequency varies across different cohorts.^2^, ^3^ Cho et al. have shown evidence of impaired central sensory processing with a circadian pattern in RLS patients.^6^ This, combined with the patient's ictal symptoms, suggests a potential overlap in the neural mechanisms underlying both RLS and focal aware seizures. Recent research has identified potential mechanistic links between epilepsy and RLS through disruption of the blood–brain barrier during seizures, which may alter central iron homeostasis, contributing to RLS development in epilepsy patients. Furthermore, hyperglutamatergic states have been implicated in both conditions as brain iron deficiency in RLS triggers a hyperglutamatergic state activating a dysfunctional cortico‐striatal‐thalamic‐cortical circuit, and nocturnal seizures enhancing glutamatergic activity, potentially exacerbating RLS symptoms.^7^, ^8^, ^9^ It has been suggested that both epilepsy and parasomnias share the activation of central pattern generators in the brain stem and spinal cord, which are neural circuits responsible for producing rhythmic, stereotypical motor behaviors. This shared activation may explain the overlap of motor symptoms in both conditions.^10^ The bidirectional relationship between these conditions suggests that optimal management of one may positively impact the other.

## CONFLICT OF INTEREST STATEMENT

The authors have no conflict of interest to disclose.

## Supporting information


Data S1.

## Data Availability

The data that support the findings of this study are available on request from the corresponding author. The data are not publicly available due to privacy or ethical restrictions.
