# Supplementary material for: Focal aware seizures manifesting as restless legs syndrome‐like symptoms in a patient with periodic limb movement disorder
Source: Epileptic Disord. 2025 May 23;27(4):678–80. doi: 10.1002/epd2.70041 (PMC12398191; doi:10.1002/epd2.70041)
Supplement: Supplementary file 1 — Data S1. [file EPD2-27-678-s001.pptx]

## Slide 1
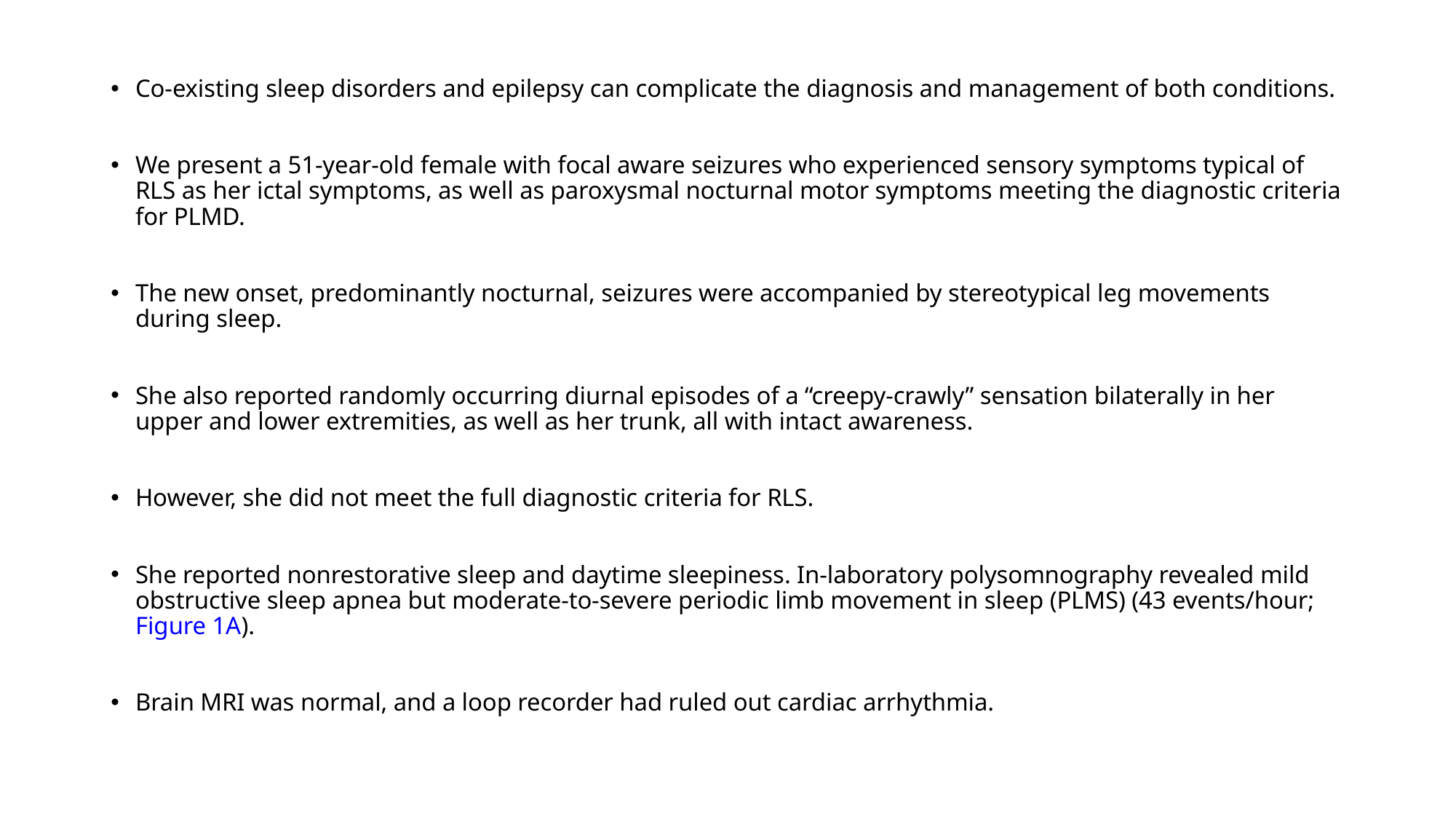

Co-existing sleep disorders and epilepsy can complicate the diagnosis and management of both conditions.
We present a 51-year-old female with focal aware seizures who experienced sensory symptoms typical of RLS as her ictal symptoms, as well as paroxysmal nocturnal motor symptoms meeting the diagnostic criteria for PLMD.
The new onset, predominantly nocturnal, seizures were accompanied by stereotypical leg movements during sleep.
She also reported randomly occurring diurnal episodes of a “creepy-crawly” sensation bilaterally in her upper and lower extremities, as well as her trunk, all with intact awareness.
However, she did not meet the full diagnostic criteria for RLS.
She reported nonrestorative sleep and daytime sleepiness. In-laboratory polysomnography revealed mild obstructive sleep apnea but moderate-to-severe periodic limb movement in sleep (PLMS) (43 events/hour; Figure 1A).
Brain MRI was normal, and a loop recorder had ruled out cardiac arrhythmia.

## Slide 2
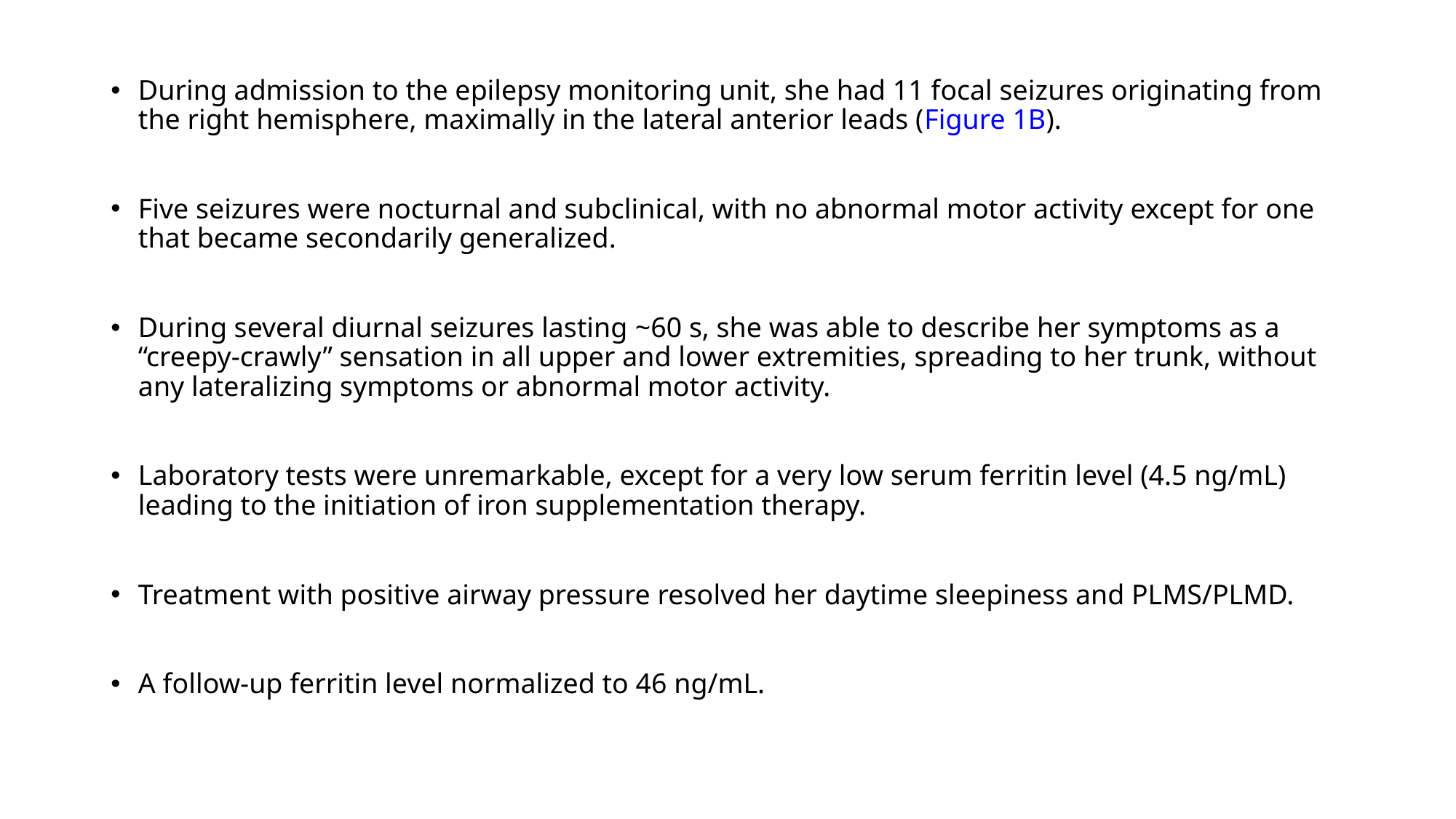

During admission to the epilepsy monitoring unit, she had 11 focal seizures originating from the right hemisphere, maximally in the lateral anterior leads (Figure 1B).
Five seizures were nocturnal and subclinical, with no abnormal motor activity except for one that became secondarily generalized.
During several diurnal seizures lasting ~60 s, she was able to describe her symptoms as a “creepy-crawly” sensation in all upper and lower extremities, spreading to her trunk, without any lateralizing symptoms or abnormal motor activity.
Laboratory tests were unremarkable, except for a very low serum ferritin level (4.5 ng/mL) leading to the initiation of iron supplementation therapy.
Treatment with positive airway pressure resolved her daytime sleepiness and PLMS/PLMD.
A follow-up ferritin level normalized to 46 ng/mL.

## Slide 3
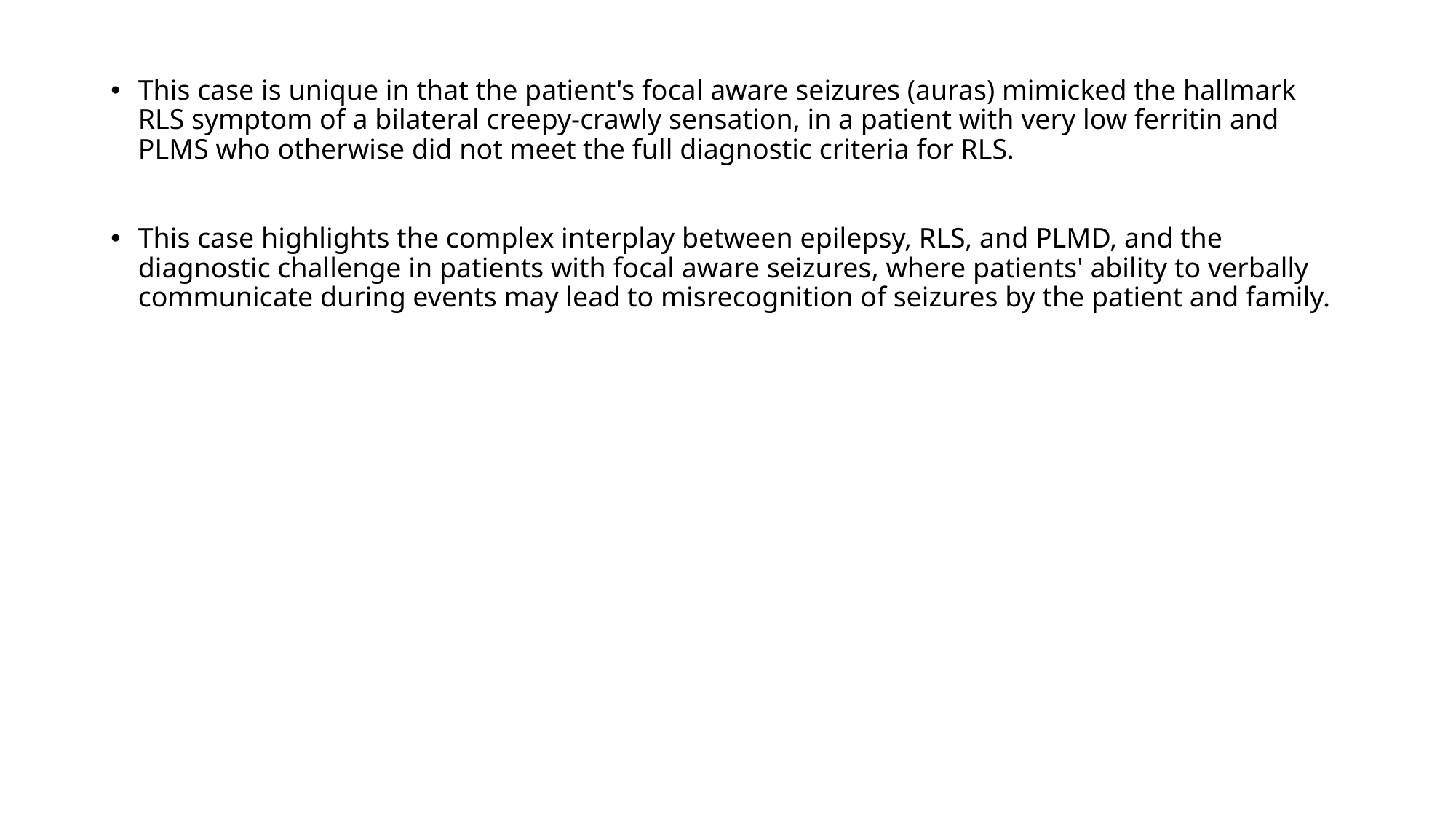

This case is unique in that the patient's focal aware seizures (auras) mimicked the hallmark RLS symptom of a bilateral creepy-crawly sensation, in a patient with very low ferritin and PLMS who otherwise did not meet the full diagnostic criteria for RLS.
This case highlights the complex interplay between epilepsy, RLS, and PLMD, and the diagnostic challenge in patients with focal aware seizures, where patients' ability to verbally communicate during events may lead to misrecognition of seizures by the patient and family.

## Slide 4
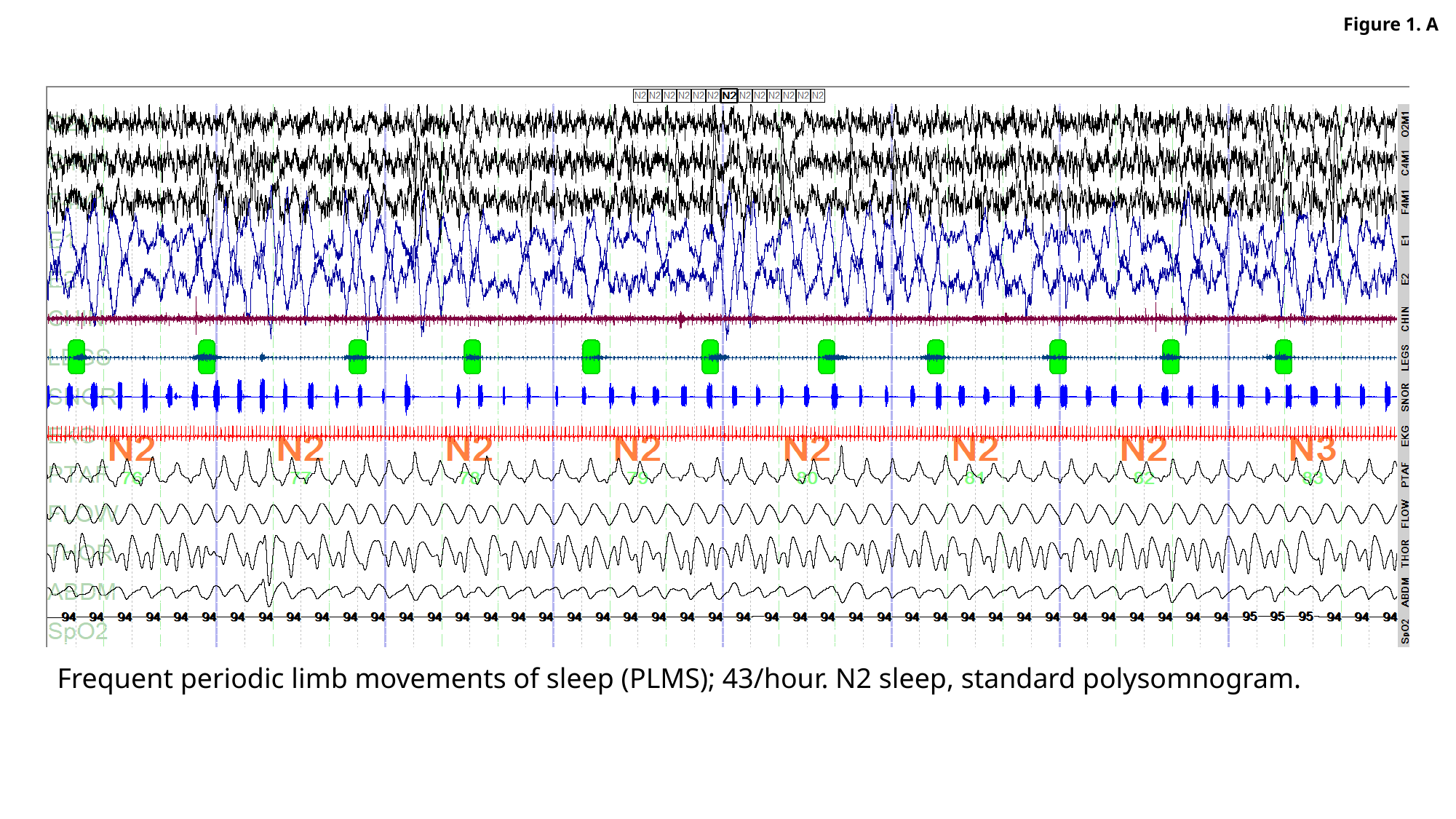

Figure 1. A
Frequent periodic limb movements of sleep (PLMS); 43/hour. N2 sleep, standard polysomnogram.

## Slide 5
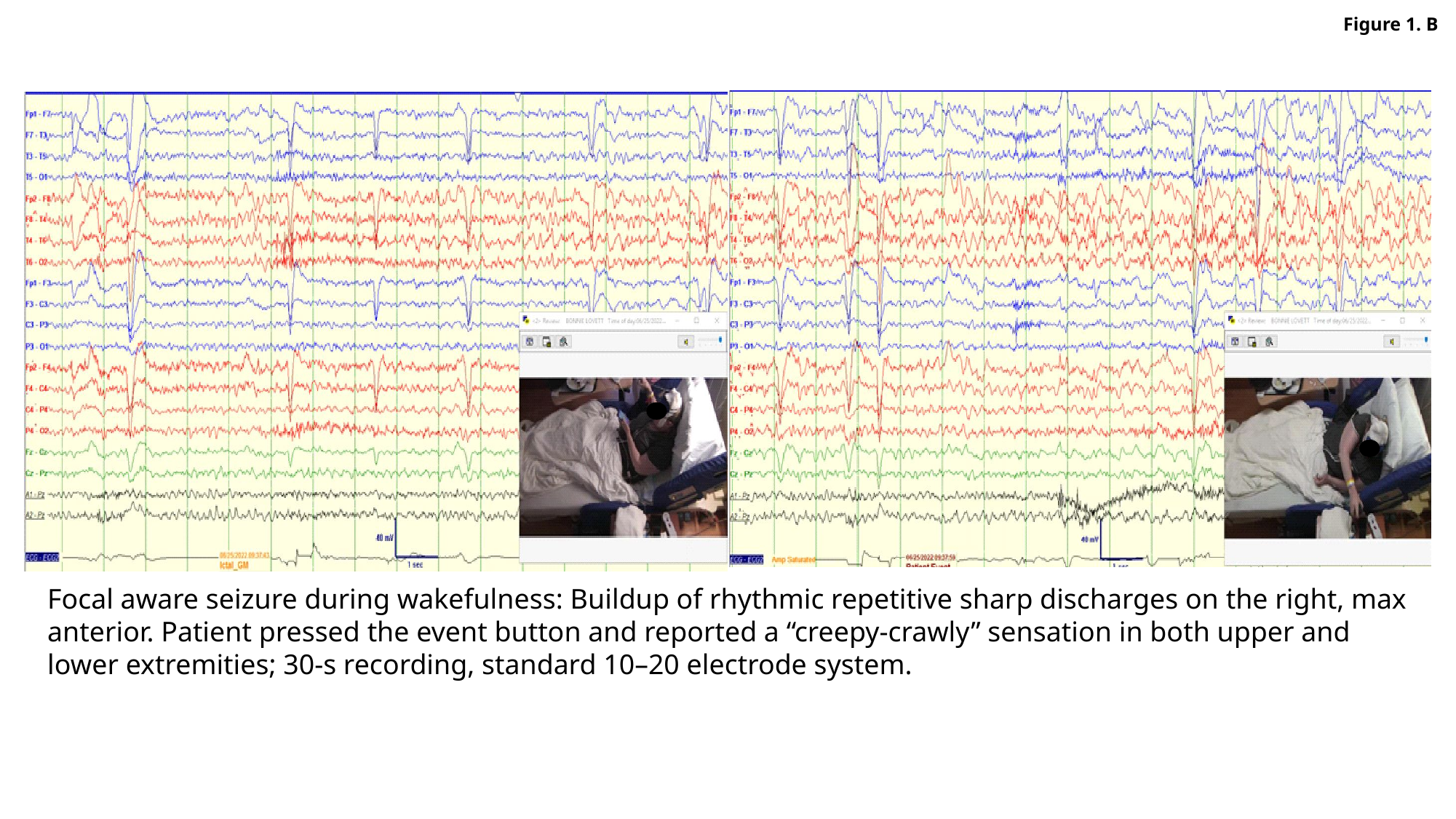

Figure 1. B
Focal aware seizure during wakefulness: Buildup of rhythmic repetitive sharp discharges on the right, max anterior. Patient pressed the event button and reported a “creepy-crawly” sensation in both upper and lower extremities; 30-s recording, standard 10–20 electrode system.
